# Editorial

**Published:** 1903-05-15

**Authors:** 


### EDITORIAL.

"THERE are two ways of treating the lad's case," said a dentist of large experience in orthodontia to the father: "One is to extract the teeth indicated the other to enlarge the arch and then force the out-of-place teeth into their normal positions."

"Which method would you recommend?"

"You say the boy 'rules the roost' both at home and abroad?"

"He does, and he'll wear what you put in to his mouth about four minutes."

"Then I must give the preference to extraction. By the other and better method I'd fail, and everybody in town would hear of it the next day; while if I succeeded, by whatever method, nobody would ever be the wiser. Better have the teeth extracted."—*Office and Laboratory.*

Doesn't it shock you to read that paragraph under its heading of "A Good Rule" and to reflect that it apparently meets the approval of the journal from which it is quoted, and other journals also which have copied it?

It shows a lack of parental control that is at least shameful if it is not actually wicked. It expresses a sort of contempt for good morals when personal comfort is involved whlch, if generally countenanced, would threaten the stability of those higher standards which make this world fit to live in, and out of which spring the motives for almost every form of benevolent action.

But what is of more interest to us is that it is a backdown from right professional standards, which if generally accepted and practiced, would bring us into deserved contempt and degrade our profession to the level of menial servitude. We can't quite understand how a professional man who has had the courage to devote himself to so useful a specialty of our profession and which involves so much the future welfare and happiness of his patients, can bring himself to the position where he will allow any ordinary circumstance to divert him from a plain duty, and, even worse, permit himself to be persuaded to do that which he knows will forever disfigure or deform his patient. The only excuse given is the fear of failure and adverse criticism. Would it not have been better for all concerned to have at least made an effort to enlist the interest and co-operation of the boy? The probabilities are that a patient and tactful man would have gained a substantial victory. which would have compensated him adequately for the struggle involved, and may be, have demonstrated to that boy that there are better things in this world than mere selfishness.

---

CALIFORNIA is the latest State to enact a new dental law. This new law contains two features which deserve attention. One is like to that of the proposed law for Illinois. It gives the State Board of Examiners power to revoke the license of any registered dentist for immoral or unprofessional conduct. It remains to be seen if such a law can or will be enforced by any State Board. Another feature which we predict will cause some trouble is a provision which compels every registered dentist to contribute two dollars annually to a fund placed in the care of the State Board, to be used in hunting out and prosecuting illegal practitioners.

---

THE editor of the *International Journal* is of the opinion that there has not been any marked improvement in the art of filling teeth in the last fifty years. This may seem somewhat ridiculous to the younger members of the profession. But there were giants in those good old times, and considering the materials and facilities with which they worked, they undoubtedly were skillful men. We are, however, of the opinion that there is on the average a higher development of skill in the profession to-day than ever before. And that in the next fifty years we shall see a still greater advance or averaging up in this direction. Fifty years do not contain time enough to settle permanently methods of practice; we are still in the experimental stages, but we shall make some advances toward uniform methods and systems of practice in the next twenty-five years, and then we shall devote our strength and time to cultivating technical art and develop skill.

